# Association between vasoactive drug use and 28-day in-hospital mortality in patients with septic shock in the intensive care unit: A retrospective study based on the MIMIC-IV database

**DOI:** 10.1097/MD.0000000000045746

**Published:** 2025-10-31

**Authors:** Qiangqiang Zhang, Yanbing Shen, Zehao Liu, Shuai Zhang, Weidong Jin, Zhonghu Li

**Affiliations:** a School of Medicine, Faculty of Medicine, Wuhan University of Science and Technology, Wuhan, Hubei, China; b General Surgery Department, General Hospital of Central Theater Command,Wuhan, Hubei, China.

**Keywords:** association analysis, intensive care units, MIMIC-IV, septic shock, vasoactive agents

## Abstract

Septic shock, a life-threatening intensive care unit condition characterized by persistent hypotension and organ dysfunction despite fluid resuscitation, is a leading cause of intensive care unit mortality. Vasoactive drugs are central to hemodynamic support in septic shock but their benefit-potential adverse event trade-off remains controversial; existing studies link high-dose vasopressors to increased mortality, though the association with 28-day mortality is unclear due to limitations like small sample sizes or single-center designs. This retrospective cohort study, based on the Medical Information Mart for Intensive Care-IV database (V3.1), included 2961 adult patients with septic shock who received vasoactive drugs and had hyperlactatemia between 2008 and 2022. The association between total vasoactive drug exposure and mortality was investigated using quartile grouping, Kaplan–Meier (K–M) survival analysis, restricted cubic spline regression, and subgroup analysis. Multivariable Cox proportional hazards models with incremental adjustments for demographics, comorbidities and laboratory markers were applied to evaluate hazard ratios. The overall in-hospital 28-day mortality rate was 31.37%. Mortality significantly increased across quartiles of vasoactive drug exposure, rising from 15.26% to 43.67% (*χ*² = 152.73, *P* < .001). Multivariable Cox models demonstrated consistently elevated mortality risks with higher exposure quartiles, showing significant dose–response trends. Restricted cubic spline analysis showed a nonlinear association between vasoactive drug exposure and 28-day mortality, with a threshold of 470.98 units: below it, lower exposure raised risk; above it, higher exposure reduced risk. Subgroup analyses found this association was more pronounced in males, patients ≤ 60 years old, those without cirrhosis, chronic kidney disease, cancer, and those with comorbid heart failure. The exposure dose of vasoactive drugs in patients with septic shock exhibits a non-linear association with 28-day mortality, with significant heterogeneity in risk characteristics across different dose ranges.

## 1. Background

Septic shock is a life-threatening condition characterized by persistent hypotension and organ dysfunction. Despite fluid resuscitation, it remains a leading cause of death in intensive care units (ICU).^[1–3]^ Despite advancements in critical care medicine, in-hospital mortality among patients with septic shock has shown limited improvement over the past few decades, with reported mortality rates ranging from 30% to 50%.^[4]^ This underscores the urgent need to identify modifiable risk factors and optimize treatment strategies to improve outcomes. Vasopressors are the cornerstone of hemodynamic support in septic shock, playing a crucial role in restoring organ perfusion.^[5]^ However, their use is associated with a complex trade-off between beneficial hemodynamic effects and potential adverse events.

Vasoactive drugs, such as norepinephrine, vasopressin, and epinephrine, are commonly used to maintain mean arterial pressure and tissue perfusion in septic shock.^[6,7]^ Norepinephrine is widely regarded as the first-line vasopressor; however, evidence suggests that the use of high-dose vasopressors is associated with increased mortality.^[8]^ Controversies remain regarding the optimal selection, dose titration, and combination strategies of vasopressors, particularly in refractory shock. Previous studies have explored the relationship between exposure to vasoactive agents and prognosis, but with inconsistent findings, often limited by small sample sizes or single-center designs.^[9]^ Furthermore, the impact of vasoactive drug kinetics, cumulative doses, and temporal patterns on 28-day mortality remains unclear.

Given the heterogeneity in clinical practice and the lack of large-scale, multicenter analyses, there is an urgent need to clarify the relationship between vasopressor use and in-hospital mortality in septic shock. This retrospective study aims to explore the association between vasoactive agent exposure and 28-day mortality in ICU patients with septic shock, using comprehensive data from the Medical Information Mart for Intensive Care IV (MIMIC-IV) database. By addressing this knowledge gap, we seek to provide evidence-based insights that can inform clinical decision-making and optimize the management of vasoactive agents in septic shock.^[10]^

## 2. Materials and methods

### 2.1. Data source

This study adopted a retrospective cohort study design, with data sourced from the MIMIC-IV (Version 3.1 database) maintained by the Laboratory for Computational Physiology at the Massachusetts Institute of Technology (MIT), USA. This project was approved by the Institutional Review Boards of MIT and Beth Israel Deaconess Medical Center. The patient information contained in the database is anonymized, and a waiver of informed consent was obtained.^[11]^ As one of the most influential public databases in the field of critical care, MIMIC-IV has undergone systematic upgrades based on its previous versions, with its data collection period extended to 2008 to 2022. This database records detailed information regarding patient demographics, laboratory tests, medications, vital signs, surgical procedures, disease diagnoses, medication administration, and follow-up survival status.^[12]^ To obtain data access, we completed the training course on protecting human research participants offered by the National Institutes of Health and passed the test of the Collaborative Institutional Training Initiative. Author’s ethical authorization ID: 13754689.

### 2.2. Inclusion and exclusion criteria methods

Inclusion criteria: adult patients (aged ≥ 18 years) who developed septic shock during ICU hospitalization; meeting the sepsis-3 diagnostic criteria, which include suspected or documented infection and an acute increase of ≥2 points in the sepsis-related organ failure assessment (SOFA) score; having used at least one common vasoactive agent during hospitalization, such as norepinephrine, dopamine, epinephrine, among others; hyperlactatemia at the initiation of vasoactive agent use (lactate level ≥ 2 mmol/L). Exclusion criteria: patients with ICU stay < 24 hours; patients with normal or missing blood lactate data; patients with missing demographic data.

### 2.3. Main endings and secondary endings

The primary outcome of the study was 28-day in-hospital all-cause mortality. The secondary endpoints were ICU mortality and mortality within 28 days after ICU admission. Information on patient mortality and the survival status of discharged patients was obtained from the U.S. Social Security Death Index.

### 2.4. Research methods

The patient data meeting the inclusion criteria were extracted from the MIMIC database using SQL language with Navicat Premium 16 data extraction software and PostgreSQL programming tools (PremiumSoft CyberTech Limited, Hong Kong, China, https://www.navicat.com).^[13]^ The extracted data underwent rigorous quality control and cleaning to ensure accuracy and completeness. The extracted patient information included: demographic information: age, gender, etc. Comorbidities: hypertension, diabetes, heart disease, etc. Vital signs: temperature, heart rate, respiratory rate, blood pressure, etc. Laboratory tests: complete blood count, biochemical indicators (e.g., hepatic and renal function, electrolytes, etc), coagulation function, inflammatory markers (e.g., C-reactive protein, procalcitonin, etc), fasting blood glucose, and triglyceride levels, among others. Vasoactive agent usage: detailed records of the types and dosages of vasoactive agents used. Blood indicators within 24 hours after ICU admission were recorded for the patients. Variables with >20% missing values were excluded.

### 2.5. Statistical analysis

All statistical analyses were performed using SPSS software (Version 22.0, IBM Corporation, Armonk) and R software (Version 4.3.2, R Foundation for Statistical Computing, Austria). For the retrospective analysis based on the MIMIC-IV database, the following statistical methods were employed: firstly, 2961 patients meeting the inclusion criteria were divided into quartile groups (Q1–Q4) according to the total exposure to vasoactive agents. Baseline characteristics were presented using descriptive statistics: continuous variables were expressed as median (interquartile range) based on their distribution characteristics, and categorical variables were presented as frequencies and percentages (%). Intergroup comparisons were conducted using the *χ*² test (for categorical variables such as gender and comorbidities, as well as outcome indicators like 28-day mortality) or corresponding nonparametric tests (for continuous variables such as age, weight, and laboratory test indicators) to analyze differences in baseline characteristics and outcomes among different exposure groups. Kaplan–Meier survival curves and the log-rank test were used to evaluate differences in 28-day in-hospital and ICU survival rates among patients in the quartile groups, visually demonstrating the association between vasoactive agent exposure and survival outcomes. Multivariable Cox proportional hazards models were further applied to evaluate independent associations, adjusting for demographics, comorbidities, laboratory values, and severity scores. Schoenfeld residuals tested proportionality assumptions. Restricted cubic spline regression models were applied to analyze the dose–response relationship between total exposure to vasoactive agents and 28-day in-hospital mortality, ICU mortality, and mortality within 28 days after ICU admission, identifying thresholds for nonlinear associations and trends in risk changes, with overall and nonlinearity tests performed. Subgroup analyses (stratified by gender, age, comorbidities, etc) were conducted to explore the heterogeneity in the association between vasoactive agents and 28-day mortality among different populations. Univariate Cox regression was used to calculate hazard ratios (HRs) and 95% confidence intervals (CIs) for each subgroup, assessing the statistical significance of interaction effects. Additionally, Cox regression models were used to further verify the correlation between vasoactive agent exposure and primary and secondary outcomes, ensuring the robustness of the results. A *P*-value < .05 was considered statistically significant.

## 3. Results

### 3.1. Characteristics of the study cohort

This study was based on Version 2.2 of the MIMIC-IV database. A total of 8268 adult patients diagnosed with septic shock who were admitted to the ICU from 2012 to 2019 were preliminarily screened. Finally, 2961 patients with septic shock meeting the study criteria were included in the final analysis. All cases had complete baseline data, records of vasoactive agent use, and a clear outcome of 28-day in-hospital mortality.

### 3.2. Results comparison of baseline data

This study included 2961 patients with septic shock from the MIMIC-IV database to analyze the correlation between vasoactive agent use and 28-day in-hospital mortality. Baseline data (e.g., demographics, SOFA score, lactate levels, vasoactive drug use) were collected at the time of septic shock diagnosis, defined as the later of the first recorded infection and the first SOFA score ≥ 2 within 24 hours of ICU admission.The results showed that the overall 28-day in-hospital mortality was 31.37%, and there was a significant increasing trend in mortality with the increase in quartile groups of total vasoactive agent exposure (i.e., dosage of vasoactive agents used) (Q1–Q4 groups: 15.26–43.67%, *χ*²=152.73, *P* < .001). Baseline characteristics indicated that patients in the high-dose exposure group (Q4) had a higher body weight (84.0 kg), significantly elevated levels of lactate (3.88 mmol/L), glucose (150.67 mg/dL), and hepatic/renal function indicators (creatinine 2.43 mg/dL, Alanine Aminotransferase (ALT) 356.09 U/L). They also had higher proportions of comorbidities such as cirrhosis (21.83%) and heart failure (43.40%), as well as significantly higher disease severity scores including Acute Physiology and Chronic Health Evaluation III, Simplified Acute Physiology Score II, and SOFA (all *P* < .001). In addition, 28-day ICU mortality also increased with the increase in agent exposure (Q1–Q4 groups: 16.21–46.09%), while there was no significant difference in in-hospital survival time among the groups (*P* = .452). These results suggest that the exposure dosage of vasoactive agents in patients with septic shock is positively correlated with 28-day mortality, and high-dose exposure may be associated with more severe metabolic disturbances, organ function impairment, and a heavier burden of comorbidities. This provides baseline evidence for further exploration of the independent association between drug dosage and prognosis (Table 1).

**Table 1:** Baseline statistical table.

Variable	Total (n = 2961)	Q1 (n = 752)	Q2 (n = 770)	Q3 (n = 701)	Q4 (n = 738)	Statistic	*P*
Demographic characteristics							
Age (Q₁, Q₃)	70.0 (59.0, 80.00)	70.00 (57.00, 81.00)	72.00 (60.00, 82.00)	69.00 (59.00, 78.00)	67.00 (56.00, 76.75)	*χ*² = 41.55	<.001
Gender, n (%)						χ² = 6.19	.103
F	1292 (43.63)	341 (46.46)	321 (44.40)	332 (43.57)	298 (40.16)		
M	1669 (56.37)	393 (53.54)	402 (55.60)	430 (56.43)	444 (59.84)		
Weight (kg)	79.10 (66.20, 95.50)	76.95 (65.31, 91.57)	77.50 (65.03, 91.00)	78.50 (65.00, 96.73)	84.00 (70.00, 100.00)	χ² = 43.37	**<.001**
Laboratory tests							
Lymphocyte count (×10⁹/L)	0.78 (0.41, 1.37)	0.75 (0.40, 1.27)	0.75 (0.41, 1.35)	0.74 (0.38, 1.34)	0.85 (0.46, 1.50)	χ² = 5.08	**.166**
Hemoglobin (g/dL)	9.95 (8.55, 11.50)	10.10 (8.60, 11.49)	9.85 (8.43, 11.46)	9.97 (8.62, 11.55)	9.93 (8.60, 11.56)	χ² = 1.53	**.676**
Red blood cells (×10¹²/L)	3.37 (2.85, 3.87)	3.42 (2.93, 3.90)	3.34 (2.83, 3.85)	3.36 (2.83, 3.84)	3.35 (2.82, 3.91)	χ² = 4.33	**.228**
White blood cells (×10⁹/L)	15.13 (9.35, 21.70)	15.84 (9.60,21.09)	15.68 (10.01,22.75)	14.34 (8.71, 20.71)	14.48 (9.35, 21.70)	χ² = 11.10	**.011**
Absolute neutrophil count (×10⁹/L)	13.46 (7.30, 20.50)	13.57 (8.07,20.96)	14.26 (7.54,21.73)	12.56 (6.26, 19.59)	13.15 (7.17, 19.36)	χ² = 3.95	**.267**
Albumin (g/dL)	2.70 (2.30, 3.10)	2.80 (2.40,3.20)	2.68 (2.30,3.10)	2.70 (2.30, 3.10)	2.60 (2.20, 3.14)	χ² = 11.51	**.009**
Calcium total (mg/dL)	7.95 (7.47, 8.47)	8.00 (7.50, 8.45)	7.91 (7.45, 8.41)	7.93 (7.43, 8.43)	7.93 (7.50, 8.59)	χ² = 4.67	**.198**
Chloride (mmol/L)	103.33 (98.60, 108.00)	104.50 (100.00, 108.50)	103.50 (98.67, 107.67)	103.00 (98.50, 108.00)	102.45 (97.45, 107.00)	χ² = 30.85	**<.001**
Potassium (mmol/L)	4.27 (3.85, 4.78)	4.13 (3.77, 4.60)	4.27 (3.85, 4.75)	4.28 (3.88, 4.80)	4.35 (3.94, 4.90)	χ² = 40.06	**<.001**
Sodium (mmol/L)	137.50 (134.25, 140.67)	138.00 (134.67, 141.00)	137.75 (134.50, 140.50)	137.08 (134.25, 140.75)	137.00 (133.50, 140.19)	χ² = 17.15	**<.001**

Alanine aminotransferase, Alt (U/L)	41.50 (21.00, 131.58)	35.00 (18.62, 87.00)	35.00 (19.00, 92.19)	44.00 (21.00, 147.38)	59.00 (24.00, 241.73)	χ² = 60.12	**<.001**
Aspartate aminotransferase, Ast (U/L)	76.00 (36.00, 233.00)	56.41 (30.12, 131.25)	61.50 (31.00, 165.00)	81.80 (38.00, 300.25)	124.00 (46.00, 466.38)	χ² = 104.52	**<.001**
Bilirubin total (mg/dL)	1.20 (0.60, 3.15)	1.02 (0.50, 2.60)	1.10 (0.60, 2.74)	1.30 (0.66, 3.34)	1.50 (0.70, 3.69)	χ² = 31.25	**<.001**
Creatinine (mg/dL)	1.63 (1.10, 2.65)	1.40 (1.00, 2.23)	1.55 (1.05, 2.46)	1.75 (1.10, 2.67)	1.93 (1.27, 3.05)	χ² = 79.15	**<.001**
Glucose (mg/dL)	141.00 (111.00, 188.19)	135.33 (107.67, 182.00)	139.00 (110.75, 181.00)	142.00 (111.00, 188.00)	150.67 (118.00, 204.00)	χ² = 26.45	**<.001**
Lactate (mmol/L)	3.15 (2.45, 4.64)	2.70 (2.27, 3.50)	3.00 (2.40, 4.03)	3.54 (2.57, 5.21)	3.88 (2.70, 5.88)	χ² = 232.46	**<.001**
Complications							
Liver cirrhosis (%)	556 (18.78%)	113 (15.40%)	111 (15.35%)	170 (22.31%)	162 (21.83%)	χ² = 21.84	**<.001**
Chronic kidney disease (%)	701 (23.67%)	163 (22.21%)	160 (22.13%)	181 (23.75%)	197 (26.55%)	χ² = 5.23	**.156**
Tumor (%)	521 (17.60%)	141 (19.21%)	145 (20.06%)	127 (16.67%)	108 (14.56%)	χ² = 9.52	**.023**
Diabetes (%)	979 (33.06%)	246 (33.51%)	228 (31.54%)	240 (31.50%)	265 (35.71%)	χ² = 4.03	**.258**
Diabetes (%)	1016 (34.31%)	207 (28.20%)	231 (31.95%)	256 (33.60%)	322 (43.40%)	χ² = 41.29	**<.001**
Coronary heart disease (%)	1048 (35.39%)	234 (31.88%)	255 (35.27%)	270 (35.43%)	289 (38.95%)	χ² = 8.07	**.045**
Disease severity score							
APACHE III	69.00 (54.00, 88.00)	57.00 (46.00, 71.75)	67.00 (53.00, 85.00)	74.00 (57.25, 91.00)	80.00 (66.00, 95.75)	χ² = 352.8	**<.001**
SAPS II	52.00 (42.00, 62.00)	44.00 (36.00, 54.00)	51.00 (43.00, 61.50)	55.00 (45.00, 65.00)	56.50 (47.00, 67.00)	χ² = 268.2	**<.001**

OASIS	40.00 (33.00, 46.00)	35.00 (29.00, 41.00)	40.00 (34.00, 46.00)	42.00 (36.00, 48.00)	43.00 (36.00, 49.00)	χ² = 280.11	**<.001**
SOFA	10.00 (7.00,12.00)	8.00 (6.00, 10.00)	9.00 (7.00, 12.00)	10.00 (8.00, 13.00)	11.00 (8.00, 13.75)	χ² = 215.07	**<.001**
Clinical outcomes							
Length of survival during hospitalization (D)	15.77 (5.32, 108.08)	19.47 (6.10, 223.18)	12.14 (3.63, 96.90)	15.04 (4.96, 81.93)	18.12 (5.54, 84.26)	χ² = 8.76#	**.033**
Hospital death (%)	252 (8.51%)	79 (10.76%)	65 (8.99%)	67 (8.79%)	41 (5.53%)	χ² = 13.56	**.004**
Death within 28 days of hospitalization (%)	929 (31.37%)	112 (15.26%)	212 (29.32%)	281 (36.88%)	324 (43.67%)	χ² = 152.73	**<.001**
Death within 28 days in ICU (%)	980 (33.10%)	119 (16.21%)	224 (30.98%)	295 (38.71%)	342 (46.09%)	χ² = 163.40	**<.001**

Bold values indicates statistically significant results.

APACHE = Acute Physiology and Chronic Health Evaluation, ICU = intensive care unit, OASIS = Organ Assessment and Support Initiative, SAPS = Simplified Acute Physiology Score.

### 3.3. Survival analysis

**Figure 1.**
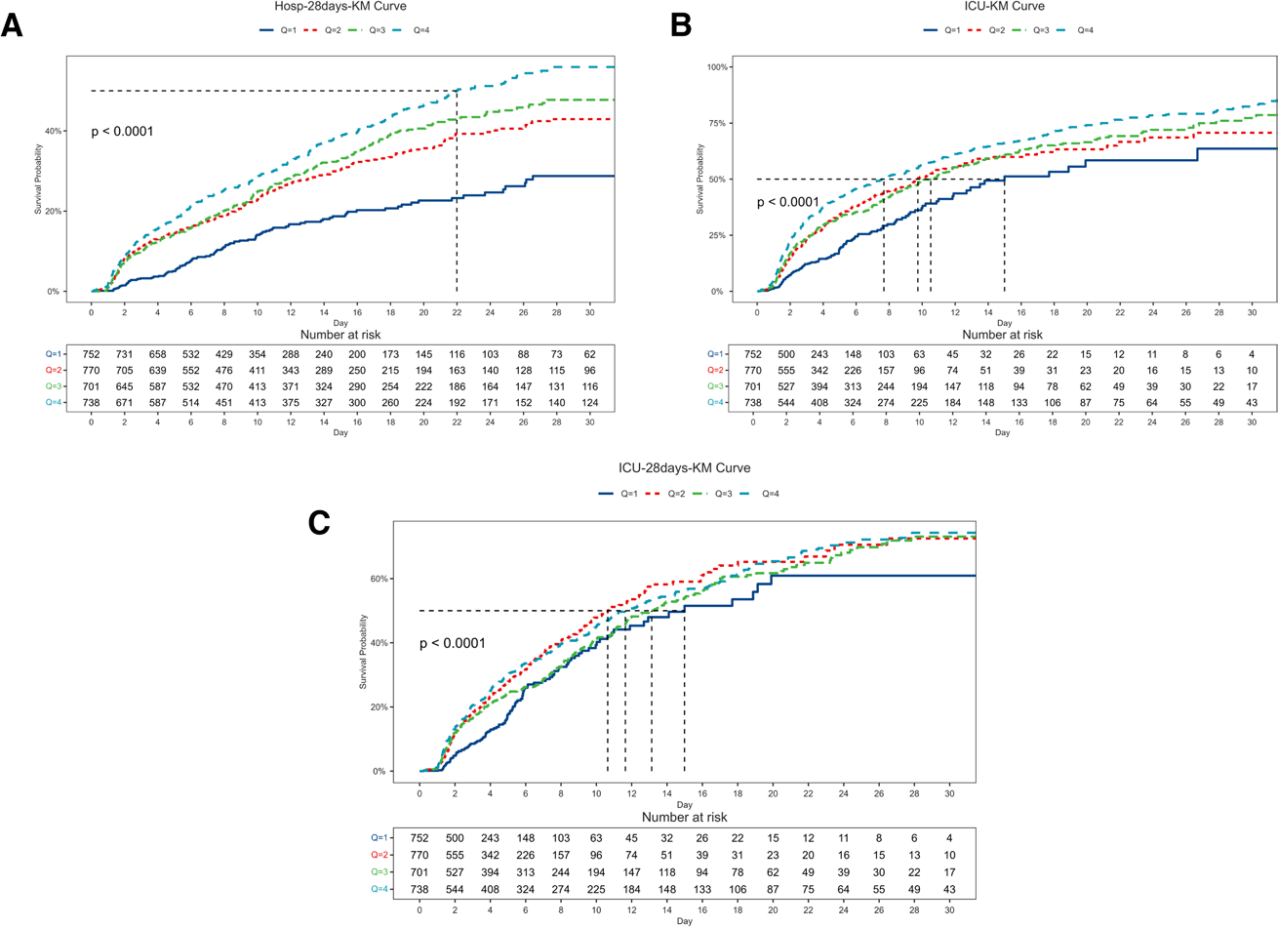
Kaplan–Meier survival curve of cumulative survival rate during hospitalization stay. (A) Kaplan–Meier survival curve of cumulative survival rate during ICU. (B) Kaplan–Meier survival curve of cumulative survival rate during 28 days ICU stay (C). ICU = intensive care unit.

A total of 2961 patients with septic shock were included and divided into quartile groups (Q1–Q4) based on the cumulative dosage of vasoactive agents (Fig. 1). The 28-day in-hospital all-cause mortality and ICU-related death outcomes were analyzed. Kaplan–Meier survival analysis showed significant differences in survival curves among the groups (all Log-rank *P* < .001). With increasing drug dosage, the 28-day survival rate of patients presented a gradient decline: the 28-day survival rate in the Q1 group (low-dose) was 84.74%, while that in the Q4 group (high-dose) was only 56.33%. The mortality rate in the high-dose exposure group increased most rapidly within 18 days after admission, and by day 28, the cumulative risk of death was 2.86 times higher than that in the Q1 group (Fig. 1A). In terms of secondary endpoints, ICU mortality and mortality within 28 days after ICU admission also showed a similar trend (Fig. 1B). The 28-day ICU mortality in the Q1 group was 16.21%, which rose to 46.09% in the Q4 group. Moreover, the mortality rate in the high-dose group reached 30% within 9 days after ICU admission, significantly higher than the 10% in the low-dose group (Fig. 1C). These results indicate that there is a clear dose-dependent association between the cumulative dosage of vasoactive agents and 28-day in-hospital all-cause mortality as well as ICU-related mortality in patients with septic shock. High-dose drug exposure, as a marker of disease severity, suggests a poorer prognosis for patients.

### 3.4. Multivariable Cox regression analysis of risk factors for 28-day in-hospital mortality

To further assess the independent association between vasoactive drug exposure and 28-day in-hospital mortality, a multivariable Cox proportional hazards regression model was developed (Table 2). The model adjusted for a comprehensive set of potential confounders, including demographic characteristics, comorbidities, key laboratory parameters, and illness severity scores. The analysis confirmed that higher quartiles of vasoactive drug exposure were independently associated with a graded increase in mortality risk. Compared to the lowest exposure quartile, Q1, the adjusted hazard ratios for Q2, Q3, and Q4 were 1.85, 2.76, and 3.40, respectively. Several other factors were also identified as significant independent predictors of mortality. Advanced age, elevated serum lactate levels, higher SOFA scores, and the presence of comorbidities including chronic kidney disease and cancer were associated with an increased risk of death. These results underscore that the positive correlation between vasoactive drug exposure and 28-day mortality remains robust after adjusting for a wide array of clinical and demographic variables.

**Table 2:** Cox proportional hazard ratios (HR) for 28-day-hospital mortality, ICU mortality, ICU 28 days mortality incidence.

Variables	Model 1		Model 2		Model 3	
	HR (95% CI)	*P*-value	HR (95% CI)	*P*-value	HR (95% CI)	*P*-value
28-day-hospital mortality						
Q1 (N = 752)	Ref.		Ref.		Ref.	
Q2 (N = 770)	2.39 [95% CI: 1.85–3.09]	<.001	2.06 [95% CI: 1.46–2.95]	.004	2.82 [95% CI: 0.98–3.92]	.34
Q3 (N = 701)	3.50 [95% CI: 2.72–4.53]	<.001	2.53 [95% CI: 1.80–3.95]	.001	1.70 [95% CI: 0.57–3.41]	.06
Q4 (N = 738)	5.94 [95% CI: 4.31–8.26]	<.001	4.03 [95% CI: 2.70–6.11]	<.001	1.45 [95% CI: 0.35–2.95]	.012
*P* for trend		<.001		<.001		.04
ICU mortality						
Q1 (N = 752)	Ref.		Ref.		Ref.	
Q2 (N = 770)	2.60 [95% CI: 1.83–3.19]	.002	2.29 [95% CI: 1.34–3.26]	.003	1.66 [95% CI: 0.64–6.04]	.30
Q3 (N = 701)	3.52 [95% CI: 2.72–4.53]	.014	3.34 [95% CI: 2.31–6.26]	.012	2.35 [95% CI: 1.90–8.26]	.05
Q4 (N = 738)	4.94 [95% CI: 4.31–7.26]	<.001	4.51 [95% CI: 2.31–9.26]	.01	4.82 [95% CI: 3.34–9.26]	.003
*P* for trend		<.001		.11		.015
ICU 28days mortality						
Q1 (N = 752)	Ref.		Ref.		Ref.	
Q2 (N = 770)	2.32 [95% CI: 1.80–2.98]	<.001	2.01 [95% CI: 1.85–3.00]	<.001	1.47 [95% CI: 1.30–4.98]	.01
Q3 (N = 701)	3.26 [95% CI: 2.56–4.17]	.003	2.48 [95% CI: 1.83–3.98]	.002	2.67 [95% CI: 1.67–4.98]	.013
Q4 (N = 738)	4.42 [95% CI: 3.46–5.64]	.001	2.95 [95% CI: 1.82–4.96]	.001	3.47 [95% CI: 2.50–5.38]	.05
*P* for trend		.03		.024		.162

Model 1 was unadjusted.

Model 2 was adjusted for sex, age, weight, height, lc, Ckd, Ca, T2DM1, Hf, IHD, and COPD.

Model 3 was adjusted for the variables in Model 2 and further adjusted for lactate, glucose, triglycerides, SOFA, APSIII, SIRS, GCS, white blood cells, calcium, sodium, pO2, pH, red blood cells, neutrophil count, and pt.

ICU = intensive care unit, T2DM = type 2 diabetes mellitus.

### 3.5. Cox regression models for all-cause mortality

**Figure 2.**
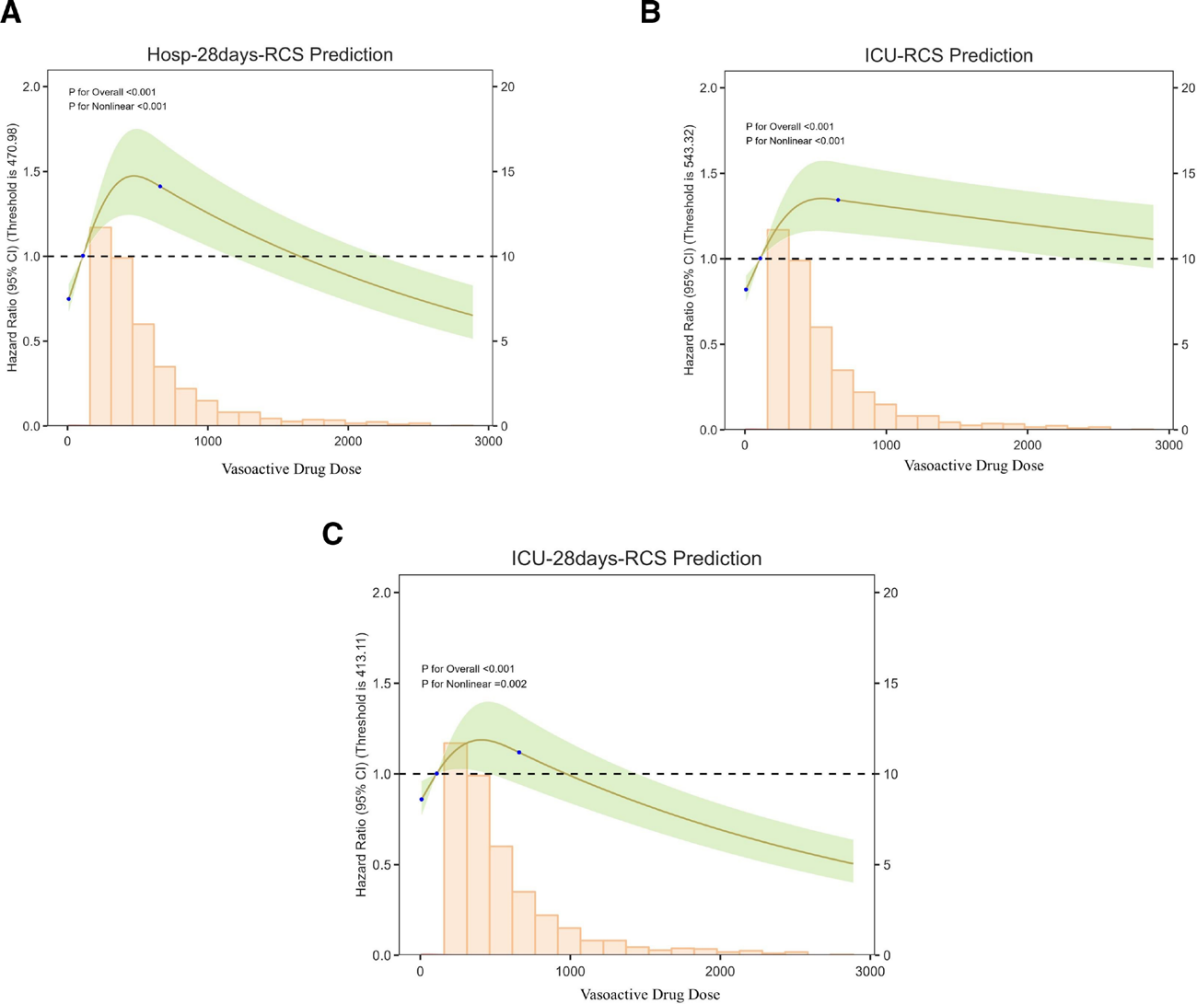
RCS regression for mortality. (A) RCS regression for in-hospital mortality in univariate analysis. (B) RCS regression for ICU mortality in univariate analysis. (C) RCS regression for ICU-28 days mortality in univariate analysis. All *P*-values for nonlinearity were <.001. ICU = intensive care unit, RCS = restricted cubic spline.

The associations between vasoactive agent use and the primary outcome (28-day in-hospital mortality) as well as secondary outcomes (ICU mortality, mortality within 28 days after ICU admission) were explored (Fig. 2). Restricted cubic spline analysis showed that, for the primary outcome, there was a nonlinear association between vasoactive agents and 28-day mortality, with a threshold of 470.98 as the risk inflection point: the risk increased in the low-value range (<470.98) and decreased in the high-value range (>470.98), with both overall and nonlinearity tests being significant (both *P*-values were *P* < .001) (Fig. 2A). For secondary outcomes: regarding ICU mortality, the vptotalval showed a unimodal nonlinear relationship, with the highest risk at the threshold of 543.32; although the risk in the high-value range decreased, it still exceeded the baseline, and both overall and nonlinearity tests were <.001 (Fig. 2B). For mortality within 28 days after ICU admission, the association with vasoactive agents presented a nonlinear trend of first increasing then decreasing, with a threshold of 413.11 as the inflection point: the risk was highest at moderate levels, and the risk in the high-value range was lower than the baseline, with the overall test <0.001 and the nonlinearity test = 0.002 (Fig. 2C). These results indicate that there are significant nonlinear associations between vasoactive agents and in-hospital as well as ICU-related mortality in patients with septic shock.

### 3.6. Subgroup analysis

**Figure 3.**
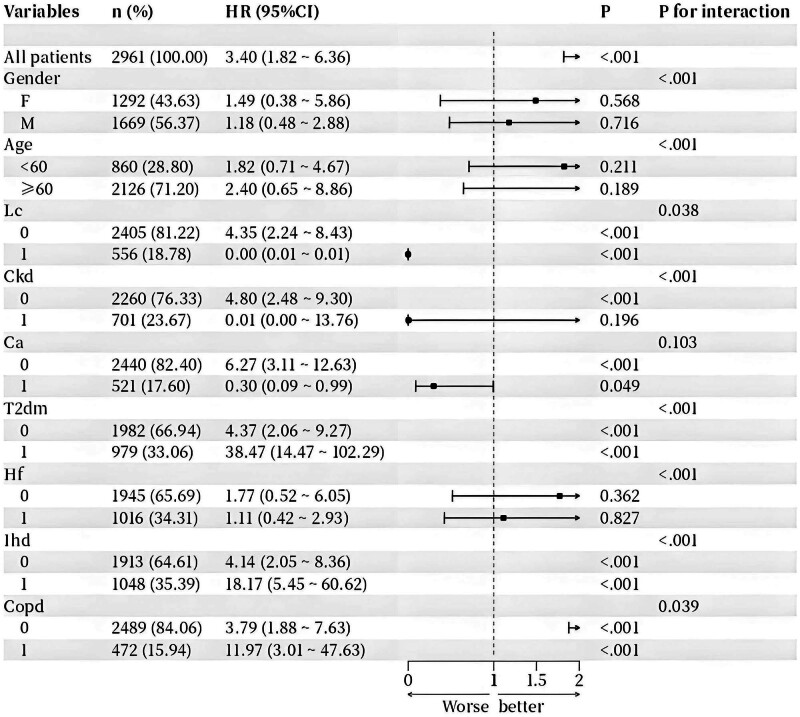
Subgroup analyses for the association between vasoactive drug use and 28-day in-hospital mortality in patients with septic shock in the intensive care unit. HR = hazard ratio.

The association between vasoactive agent use and 28-day in-hospital mortality was explored. Overall analysis revealed a significant positive correlation between vasoactive agent use and mortality (HR = 3.40, 95% CI: 1.82–6.36, *P* < .001) (Fig. 3). However, subgroup analysis uncovered significant heterogeneity (*P* for interaction < .001), indicating distinct differences in risk associations among patients with varying baseline characteristics. Notably, patients with specific comorbidities exhibited extremely high mortality risk: the risk surged in those with type 2 diabetes mellitus (HR = 38.47, 95% CI: 14.47–102.29), while patients with ischemic heart disease (HR = 18.17, 95% CI: 5.45–60.62) and chronic obstructive pulmonary disease (HR = 11.97, 95% CI: 3.01–47.63) also faced significantly elevated mortality risk. In contrast, in subgroups of patients with liver disease (Lc), chronic kidney disease, and cancer (Ca), vasoactive agents showed a potential trend toward survival benefit (Lc: HR = 0.00; Ckd: HR = 0.01; Ca: HR = 0.30, all *P* < .001). Furthermore, although statistical significance was observed in subgroups stratified by gender, age, and heart failure (Hf), their hazard ratios were close to or crossed the null value (e.g., male: HR = 1.18, 95% CI: 0.48–2.88; age ≥ 60 years: HR = 2.40, 95% CI: 0.65–8.86; Hf: HR = 1.11, 95% CI: 0.42–2.93), indicating relatively weak association strength within these subgroups. In summary, the impact of vasoactive agents on the prognosis of patients with septic shock is highly dependent on the status of underlying diseases, with the strongest associations particularly prominent in patients with type 2 diabetes mellitus, ischemic heart disease, and chronic obstructive pulmonary disease, highlighting the necessity of individualized risk assessment (Fig. 3).

## 4. Discussion

This study is the first to systematically investigate the relationship between the exposure dose of vasoactive agents and 28-day in-hospital mortality in patients with septic shock. The results showed a significant positive correlation between the total dose of vasoactive agents and mortality, and this correlation remained statistically significant after adjusting for demographic characteristics, comorbidities, and disease severity. This finding expands the understanding of the application of vasoactive agents in critical care: the dose of vasoactive agents is not only a marker of hemodynamic support but also can serve as an independent indicator for prognostic assessment. In recent years, although previous studies have suggested that high-dose vasoactive agent use is associated with poor prognosis in patients with septic shock, conclusions have remained controversial due to limitations such as small sample sizes or single-center designs.^[14,15]^ Through a large-sample analysis of the MIMIC-IV database, this study confirms that each increase in the quartile of vasoactive agent dose is associated with a 2.86-fold increase in 28-day in-hospital mortality, which is consistent with the report by Yang et al that vasopressor exposure is independently associated with ICU mortality.^[16]^ Furthermore, lactate levels, indicators of liver and kidney function impairment, and SOFA scores were significantly elevated in patients in the high-dose group, indicating that drug dosage may reflect the severity of the disease rather than merely drug toxicity.^[17]^ It is indicated that the dosage of vasoactive drugs is closely related to the severity and prognosis of septic shock.^[18]^ Septic shock can lead to circulatory failure and multiple organ dysfunction, and vasoactive agents are crucial for maintaining organ perfusion. However, the positive correlation between drug dosage and mortality may reflect the phenomenon of “treatment confounding by disease severity”: meaning that patients with more severe conditions require higher doses.^[19–21]^ The dosage of vasoactive agents can serve as a surrogate indicator for assessing the severity of septic shock, providing a reference for clinicians to rapidly evaluate prognosis.^[14,22,23]^

The exact biological mechanisms underlying the relationship between the dosage of vasoactive agents and the prognosis of septic shock remain unclear. While improving hemodynamics, vasoactive agents may also induce endothelial dysfunction, oxidative stress, and microcirculatory disturbances, thereby exacerbating organ damage.^[24]^ Based on baseline data, we observed significant differences in Acute Physiology and Chronic Health Evaluation III and SOFA scores among patients in different vasoactive agent dosage groups, indicating that drug dosage is closely associated with disease severity. Additionally, the use of high-dose vasoactive agents may be related to refractory shock, which is often accompanied by severe inflammatory responses and metabolic disturbances, further affecting patient prognosis. Sensitivity analysis showed that the linear relationship between vasoactive agent dosage and in-hospital mortality remained consistent in subgroups of younger patients, females, patients with lower body weight, and those without cardiovascular disease. This result may be attributed to the fact that advanced age, male gender, and cardiovascular comorbidities are traditional risk factors for poor prognosis in septic shock, which may mask the association between drug dosage and mortality in specific populations. Cox regression analysis further confirmed the consistent relationship between vasoactive agent dosage and 28-day ICU mortality, supporting the robustness of the results.

However, it is important to recognize the limitations of this study. First, as a retrospective observational study, it cannot establish a causal relationship between vasoactive agent dosage and mortality, and there may be reverse causality (i.e., patients with more severe conditions require higher doses). Second, the types of vasoactive agents and administration methods (continuous vs intermittent) were not dynamically monitored, and the study only focused on total dosage, which may not fully reflect the impact of pharmacokinetics on outcomes. Third, some confounding factors, such as microcirculatory parameters, nutritional status, and specific infection types, were not fully considered and may have influenced the results. Fourth, changes in sepsis diagnostic criteria and treatment guidelines during the study period (2008–2019) may have introduced selection bias. Fifth, the impact of concomitant use of other cardiovascular drugs (e.g., inotropes) on the efficacy of vasoactive agents was not evaluated.

Nevertheless, we conducted rigorous sensitivity analyses using multivariable Cox regression models with incremental adjustments for demographics, comorbidities, and laboratory markers. The consistent and graded increase in mortality risk across vasoactive drug exposure quartiles in all 3 models reinforces the robustness of our primary findings. Although the observed associations may still be influenced by the limitations mentioned above, the stability of the risk estimates across progressively adjusted models suggests that the core conclusion is methodologically sound.

Further prospective, multicenter studies are warranted to validate these findings, ideally incorporating detailed pharmacokinetic monitoring, standardized dosing protocols, and comprehensive biomarker profiling to better elucidate the causal relationship between vasoactive drug exposure and outcomes in septic shock.

## 5. Conclusion

This study confirms that there is a significant positive correlation between the exposure dose of vasoactive agents and 28-day in-hospital mortality in patients with septic shock, with the risk of death in the population receiving high-dose administration being 2.86 times higher than that in the lowest dose group. Future prospective studies are needed to further verify the optimal dosage range of vasoactive agents and explore their interaction mechanisms with microcirculatory perfusion and inflammatory regulation, so as to guide clinical precise hemodynamic management.

## Author contributions

**Conceptualization:** Qiangqiang Zhang, Zehao Liu, Weidong Jin.

**Data curation:** Qiangqiang Zhang, Shuai Zhang.

**Formal analysis:** Shuai Zhang.

**Investigation:** Yanbing Shen.

**Methodology:** Yanbing Shen.

**Software:** Qiangqiang Zhang.

**Validation:** Qiangqiang Zhang, Yanbing Shen.

**Visualization:** Yanbing Shen, Shuai Zhang.

**Writing – original draft:** Qiangqiang Zhang.

**Writing – review & editing:** Yanbing Shen, Weidong Jin, Zhonghu Li.
